# A New In-Flight Alignment Method with an Application to the Low-Cost SINS/GPS Integrated Navigation System

**DOI:** 10.3390/s20020512

**Published:** 2020-01-16

**Authors:** Zhenglong Lu, Jie Li, Xi Zhang, Kaiqiang Feng, Xiaokai Wei, Debiao Zhang, Jing Mi, Yang Liu

**Affiliations:** 1 National Key Laboratory for Electronic Measurement Technology, North University of China, Taiyuan 030051, China; s1706129@st.nuc.edu.cn (Z.L.); b1506011@st.nuc.edu.cn (K.F.); b1806023@st.nuc.edu.cn (X.W.); b1706012@st.nuc.edu.cn (D.Z.); s1806038@st.nuc.edu.cn (J.M.); s1806125@st.nuc.edu.cn (Y.L.); 2 Key Laboratory of Instrumentation Science & Dynamic Measurement, North University of China, Taiyuan 030051, China; 3 School of Electrical Control Engineering, North University of China, Taiyuan 030051, China; zhangxi@nuc.edu.cn

**Keywords:** in-flight alignment, integrated navigation system, optimization-based alignment

## Abstract

The optimization-based alignment (OBA) methods, which are implemented by the optimal attitude estimation using vector observations—also called double-vectors—have proven to be effective at solving the in-flight alignment (IFA) problem. However, the traditional OBA methods are not applicable for the low-cost strap-down inertial navigation system (SINS) since the error of double-vectors will be accumulated over time due to the substantial drift of micro-electronic- mechanical system (MEMS) gyroscope. Moreover, the existing optimal estimation method is subject to a large computation burden, which results in a low alignment speed. To address these issues, in this article we propose a new fast IFA method based on modified double-vectors construction and the gradient descent method. To be specific, the modified construction method is implemented by reducing the integration interval and identifying the gyroscope bias during the construction procedure, which improves the accuracy of double-vectors and IFA; the gradient descent scheme is adopted to estimate the optimal attitude of alignment without complex matrix operation, which results in the improvement of alignment speed. The effect of different sizes of mini-batch on the performance of the gradient descent method is also discussed. Extensive simulations and vehicle experiments demonstrate that the proposed method has better accuracy and faster alignment speed than the related traditional methods for the low-cost SINS/global positioning system (GPS) integrated navigation system

## 1. Introduction

The strap-down inertial navigation system (SINS) has been widely used in the determination of attitude, velocity and position of an object based on the dead-reckoning by making use of the measurements provided by the inertial measurement unit (IMU) [1,2,3]. To reduce the long-time navigation error mainly caused by the bias of accelerometers and gyroscopes, the SINS is often integrated with the global positioning system (GPS), which constructs the SINS/GPS integrated navigation system [4,5,6,7,8]. The heart of guaranteeing the performance of SINS is to accomplish the initial alignment and obtain an accurate initial condition [9,10]. In the SINS/GPS integrated navigation system, the initial velocity and position can usually be obtained directly from GPS. Therefore the main aim of initial alignment is to determine the initial attitude between the body frame and reference navigation frame [11]. In recent years, many attitude determination methods have been proposed for the initial attitude alignment, for example, analytic alignment method, transfer alignment method [12,13,14,15,16]. However, they are not applicable when the carrier is in-flight, which is the main focus of this paper. 

To address the in-flight alignment of GPS-aided SINS, the optimization-based alignment (OBA) method has been proposed for high accuracy SINS [17,18,19,20,21,22]. The OBA method is derived based on the idea of attitude matrix decomposition, where the real-time attitude matrix is decomposed into three parts—two time-varying matrices which are respectively the attitude change of the body frame and the navigation frame and one constant matrix which is the objective attitude matrix of the alignment [23]. The vector observations can be constructed with the former two matrices to calculate the objective matrix [24,25,26,27,28,29]. The heart of the OBA method is how to construct the vector observations and calculate the optimal alignment matrix [23,30]. For the construction procedure, the OBA method constructs the double-vectors as vector observations based on the traditional velocity/position integration formula by making use of the measurements provided by the outputs of accelerometer, gyroscope and GPS [30]. It has been proven that the above-mentioned construction method can achieve high enough accuracy for the computation of double-vector in IFA [31]. However, the construction method is not applicable for the low-cost SINS since the low-cost IMU contains substantial bias, which will be integrated with time during the construction procedure and leading to the large calculation error in double-vectors. For the optimal estimation procedure of the alignment matrix, the IFA can be regarded as Wahba’s problem, which is aimed to minimize the loss function constructed by double-vectors [32]. To solve the problem, many optimization-based methods have been proposed, among which the most representative ones include singular value decomposition (SVD), TRIAD and quaternion estimator (QUEST). The SVD method can obtain the objective attitude by decomposing the matrix, which is constructed with the cross-product of the sequence of double-vectors, into one diagonal matrix and two unit-orthogonal matrices. The product of the latter two matrices is exactly the objective matrix [25]. However, this matrix operation is very complicated and computationally expensive. TRIAD is a simple and effective estimation method and the objective matrix can be obtained by the product of two matrices which are respectively constructed by the cross-product of two double-vectors [33]. However, it has low accuracy and poor robustness because only two observations are used in each estimation [24]. Davenport’s *q*-method derives the quaternion form of Wahba’s problem [34]. The optimal quaternion can be obtained by an eigenvalue equation. To simplify the solution, the QUEST method, which is developed from Davenport’s *q*-method and the most adopted in practical use, transforms the solution of the eigenvalue equation in Davenport’s *q*-method into a solution of the quartic equation [35]. However, the equation is still complicated to build and solve for low-cost processors. According to the aforementioned analysis, the traditional IFA method cannot be performed well on the low-cost integration navigation systems. So a fast, accurate and robust IFA method needs to be designed for low-cost SINS/GPS integration navigation systems and this is also the main research content of this article.

In this paper, we propose a new in-flight alignment method with an application to the low-cost SINS/GPS integrated navigation system. The main contributions of our research are twofold—(1) a modified construction method is proposed to construct the double-vectors more accurately. In this method, the integration interval of construction is shorter than the traditional method, so the accelerometer bias is suppressed, and the error will not increasingly accumulate with time. A linear Kalman filter model is adopted to identify the bias of gyroscope for improving the accuracy of double-vectors. (2) A fast IFA method based on gradient descent is designed to improve the performance of the optimal estimation method and make it available on the low-cost system. It can obtain the initial attitude directly and does not involve any complex matrix operation, so the proposed method is very efficient and easy to implement. Verified by the simulations, this method can become faster or more accurate and robust to meet different usage needs by adjusting the size of the mini-batch. The simulations and experiments are implemented to compare the performance of different IFA algorithms and the results show that the proposed method is superior to traditional methods.

The rest of this paper is organized as follows. The traditional OBA methods and their problems of the application on low-cost systems are formulated in Section 2. The proposed method consisting of the modified double-vectors construction method and the fast IFA method based on gradient descent is detailed in Section 3. The results of simulations and experiments are used to verify the performance of the proposed method and shown in Section 4. Finally, the article is concluded in Section 5.

## 2. Problem Formulation

### 2.1. Preliminary

The local geographical coordinate is selected as the navigation frame denoted by *n* and the inertial measurement unit (IMU) coordinate frame fixed on the carrier as the body frame denoted by *b*. Freezing the navigation frame and body frame in the inertial frame denoted by I at the initial time, they are respectively renamed by the inertial navigation frame denoted by *in* and the inertial body frame denoted by *ib*. These two frames are stationary in the *i* frame and respectively coincident with the *n* frame and *b* frame at the initial moment. In this article, the direction cosine matrix (after this referred to as the attitude matrix) is used for representing the transformation between two frames which are respectively denoted by the superscripts and the subscripts of the attitude matrix. According to the chain rule of the attitude matrix, the real-time attitude matrix $C_{n\left(t\right)}^{b\left(t\right)}$ from *n* frame to *b* frame can be written as [11]

(1) $$C_{n\left(t\right)}^{b\left(t\right)}=C_{ib}^{b\left(t\right)}C_{in}^{ib}C_{n\left(t\right)}^{in},$$

where $C_{ib}^{b\left(t\right)}$ is the real-time attitude matrix from *ib* frame to current *b* frame, $C_{in}^{ib}$ is the constant attitude matrix from *in* frame to *ib* frame, $C_{n\left(t\right)}^{in}$ is the real-time attitude matrix from the current *n* frame to the *in* frame. $C_{ib}^{b\left(t\right)}$ can be calculated as follows

(2) $${\dot{C}}_{ib}^{b(t)}=-\left(\omega_{ib}^{b}\times \right)C_{ib}^{b(t)},$$

where $\omega_{ib}^{b}$ is the body angular rate that can be obtained by gyroscopes. The operation (⋅×) is the cross-product matrix. The value of the $C_{ib}^{b\left(t\right)}$ can be calculated by many commonly used methods and the details are omitted for brevity [36]. According to the differential equation of the attitude matrix, $C_{in}^{n\left(t\right)}$ can be calculated as [37]

(3) $$C_{n(t)}^{in}=\left[\begin{array}{ccc} \cos\Delta \lambda & -\sin L_{\left(t\right)}\sin\Delta \lambda & \cos L_{\left(t\right)}\sin\Delta \lambda \\ \sin L_{in}\sin\Delta \lambda & \sin L_{in}\sin L_{\left(t\right)}\cos\Delta \lambda +\cos L_{in}\cos L_{\left(t\right)} & -\sin L_{in}\cos L_{\left(t\right)}\cos\Delta \lambda +\cos L_{in}\sin L_{\left(t\right)}\\ -\cos L_{in}\sin\Delta \lambda & -\cos L_{in}\sin L_{\left(t\right)}\cos\Delta \lambda +\sin L_{in}\cos L_{\left(t\right)} & \cos L_{in}\cos L_{\left(t\right)}\cos\Delta \lambda +\sin L_{in}\sin L_{\left(t\right)} \end{array}\right]$$

where $\lambda_{in}$ and $L_{in}$ are the longitude and latitude at the initial moment of alignment respectively; $\lambda_{\left(t\right)}$ and $L_{\left(t\right)}$ are the longitude and latitude of the current local position respectively; the increment of longitude $\Delta \lambda =\lambda_{\left(t\right)}-\lambda_{in}+\omega_{ie}^{n}t$, where $\omega_{ie}^{n}$ is a constant representing the earth rotation rate. The longitude and latitude can be measured by GPS, so $C_{n\left(t\right)}^{in}$ can be determined according to (3). Because the constant matrix $C_{in}^{ib}$ is the only unknown item on the right side of the equal sign of (1), the current attitude $C_{n\left(t\right)}^{b\left(t\right)}$ can be determined by the constant matrix $C_{in}^{ib}$. Therefore, the key to IFA is how to get the accurate value $C_{in}^{ib}$ which is also called the objective matrix.

### 2.2. Traditional Optimization-based Alignment Method

According to (1), The vector observations in *n* frame and *b* frame can be transformed into that in *in* frame and *ib* frame by $C_{n\left(t\right)}^{in}$ and $C_{ib}^{b\left(t\right)}$, which is called double-vectors. And they can be constructed infinitely as long as the measurement of inertial sensors and GPS is valid. For each pair of double-vectors, they can only be converted to each other by a unique constant matrix, which is also the objective matrix. So it can be calculated by optimal estimation methods. Traditional OBA methods have two procedures. The first is constructing the double-vectors in *in* frame and *ib* frame with the information measured by inertial sensors and GPS; The second is the optimal estimation of the initial attitude using double-vectors. In this section, the traditional construction method and optimal estimation method are introduced in detail.

To construct the double-vectors, The specific force equation is introduced as

(4) $${\dot{v}}^{n}=f^{n}-\left(2\omega_{ie}^{n}+\omega_{en}^{n}\right)\times v^{n}+g^{n},$$

where $v^{n}$ is the velocity can be obtained by GPS of the carrier, $f^{n}$ is the specific force of carrier, $g^{n}$ is the gravity vector, $\omega_{en}^{n}$ is the angular rate from *e* frame to *n* frame and $\omega_{ie}^{n}$ is the earth rotation rate. The superscript *n* represents that they are measured in the *n* frame. $\omega_{en}^{n}$ and $\omega_{ie}^{n}$ can be calculated as follows

(5) $$\left\{ \begin{array}{l} \omega_{ie}^{n}=\left[\begin{array}{ccc} 0 & \omega_{ie}^{i}\cos L & \omega_{ie}^{i}\sin L \end{array}\right]\\ \omega_{en}^{n}=\left[\begin{array}{ccc} -\frac{v_{N}}{R_{M}} & \frac{v_{E}}{R_{N}} & \frac{v_{E}}{R_{N}}\tan L \end{array}\right] \end{array}\right.,$$

where $v_{E}$ and $v_{N}$ are respectively the east and north velocity in the n frame, $R_{M}$ and $R_{N}$ are the radius of the prime vertical circle and meridian circle respectively [29]. According to (1), the specific force equation can be written as

(6) $${\dot{v}}^{n}=C_{in}^{n}C_{ib}^{in}C_{b}^{ib}f^{b}-\left(2\omega_{ie}^{n}+\omega_{en}^{n}\right)\times v^{n}+g^{n},$$

where $f^{b}$ is the specific force in the *b* frame (6) can be rewritten as

(7) $$C_{in}^{ib}C_{n}^{in}\left({\dot{v}}^{n}+\left(2\omega_{ie}^{n}+\omega_{en}^{n}\right)\times v^{n}-g^{n}\right)=C_{b}^{ib}f^{b}.$$

$C_{b}^{ib}$ and $C_{n}^{in}$ can be obtained according to (2) and (3), $\omega_{en}^{n}$ and $\omega_{ie}^{n}$ can be calculated by the velocity and position information provided by GPS according to (5), $f^{b}$ can be measured by the accelerometer of SINS. To avoid the velocity differential error, both sides of (7) are integrated for constructing the vector observations [30]. It is given by

(8) $$\alpha \left(t\right)=C_{in}^{ib}\beta \left(t\right),$$

where

(9) $$\left\{ \begin{array}{l} \alpha \left(t\right)=\int_{0}^{t}C_{b\left(t\right)}^{ib}f^{b}dt\\ \beta \left(t\right)=C_{n\left(t\right)}^{in}v^{n}-v^{n}\left(0\right)+\int_{0}^{t}C_{n\left(t\right)}^{in}\left(2\omega_{ie}^{n}+\omega_{en}^{n}\right)\times v^{n}dt-\int_{0}^{t}C_{n\left(t\right)}^{in}g^{n}dt \end{array}\right..$$

α and β are commonly called double-vector [38]. It can be seen from (9) that ${\dot{v}}^{n}$ is replaced by $v^{n}$ which can be measured by GPS directly. Many pairs of double-vectors can be obtained over time through (9) and only the objective matrix $C_{in}^{ib}$ can satisfy the relationship as (8). So $C_{in}^{ib}$ can be obtained by the optimal estimation method.

For the optimal estimation of the objective attitude, it can be regarded as a Wahba’s problem. The optimal $C_{in}^{ib}$ can be obtained by minimizing the loss function *J* of the Wahba’s problem as [32]

(10) $$J\left(C_{in}^{ib}\right)=\frac{1}{2}\sum_{k=1}^{N}s_{k}{\left\|\alpha_{k}-C_{in}^{ib}\beta_{k}\right\|}^{2},$$

where *N* is the number of double-vectors, k=1,2,⋯,M, $s_{k}$ are positive weight and $\sum_{k=1}^{N}s_{k}=1$. Wahba’s problem is obviously a least-squares problem. Many mathematical methods have been proposed to solve it and the most contributing is Davenport’s q-method [34]. In this method, the loss function is expanded as

(11) $$J\left(C_{in}^{ib}\right)=\frac{1}{2}\sum_{k=1}^{N}s_{k}{\left[\alpha_{k}-C_{in}^{ib}\beta_{k}\right]}^{T}\left[\alpha_{k}-C_{in}^{ib}\beta_{k}\right]=1-\sum_{k=1}^{N}\left[s_{k}\alpha_{k}{}^{T}C_{in}^{ib}\beta_{k}\right].$$

Defining the gain function $g\left(C_{in}^{ib}\right)$ as

(12) $$g\left(C_{in}^{ib}\right)=\sum_{k=1}^{N}\left[s_{k}\alpha_{k}{}^{T}C_{in}^{ib}\beta_{k}\right]=tr\left[C_{in}^{ib}B^{T}\right],$$

with *B* given by

(13) $$B=\sum_{k=1}^{N}s_{k}\alpha_{k}\beta_{k}^{T}.$$

It can be seen that minimizing $J\left(C_{in}^{ib}\right)$ can be achieved by maximizing $g\left(C_{in}^{ib}\right)$. Using quaternion *q* instead of attitude matrix, $C_{in}^{ib}$ can be regarded as the function of *q* as

(14) $$C_{in}^{ib}\left(q\right)=\left(\eta^{2}-\varepsilon^{T}\varepsilon \right)I_{3\times 3}+2\varepsilon \varepsilon^{T}-2\eta \varepsilon \times ,$$

where $q={\left[\begin{array}{cc} \eta & \varepsilon \end{array}\right]}^{T}$, η is the scalar part and ε is the vector part are respectively the scalar and vector part of the quaternion *and* $\bar{q}={\left[\begin{array}{cc} \varepsilon & \eta \end{array}\right]}^{T}$. Substituting (14) into (12), the gain function in the quaternion form is as

(15) $$g\left(\bar{q}\right)=\sigma \left(\eta^{2}-\varepsilon^{T}\varepsilon \right)I_{3\times 3}+\varepsilon^{T}S\varepsilon +2\eta \varepsilon^{T}Z={\bar{q}}^{T}K\bar{q},$$

where

(16) $$\sigma =tr\left(B\right)=tr\left(B^{T}\right),\;S=B+B^{T},\;Z=\sum_{k=1}^{N}s_{k}\left(\alpha_{k}\times \beta_{k}\right),\;K=\left[\begin{array}{cc} S-\sigma I & Z_{1\times 3}\\ Z_{3\times 1}^{T} & \sigma \end{array}\right].$$

$g\left(\bar{q}\right)$ can be maximized by the optimal quaternion of IFA. Considering the constraint ${\bar{q}}^{T}\bar{q}=1$, the Lagrange multiplier λ can be introduced to reconstruct the loss function as

(17) $$g^{\prime }\left(\bar{q}\right)={\bar{q}}^{T}K\bar{q}-\lambda \left({\bar{q}}^{T}\bar{q}-1\right),$$

where $g^{\prime }\left(\bar{q}\right)$ is the new gain function with the constraint of the quaternion feature. Set its derivative to 0 to find the max value of the gain function and yields

(18) $$K\bar{q}=\lambda \bar{q}.$$

It can be clearly seen that λ is an eigenvalue of *K* and the optimal quaternion ${\bar{q}}_{opt}$ is the eigenvector corresponding to the largest eigenvalue. It is a very complex process to calculate the eigenvector of the matrix [34]. To simplify the computation, the QUEST method which is developed from Davenport’s method derives a quartic equation about λ as

(19) $$\lambda^{4}-\left(a+b\right)\lambda^{2}-c\lambda +\left(ab+c\sigma -d\right)=0,$$

where

(20) $$\begin{array}{c} a=\sigma^{2}-\mathrm{tr}(\mathrm{adj}\;S),\;b=\sigma^{2}+Z^{T}Z,\\ c=\det\;S+Z^{T}SZ,\;d=Z^{T}S^{2}Z \end{array}$$

where the tr (⋅) is the trace of the matrix, adj (⋅) is the adjoint matrix and det (⋅) is the determinant of the matrix. The max solution of the equation is also the max eigenvector of *K* [24]. However, the construction and solution of the eigenvalue are still very complicated. The computation module of low-cost navigation systems may not be able to afford such a large amount of computation.

### 2.3. Problem Formulation

The traditional OBA method requires high sensor accuracy and computing power. However, The low-cost integrated navigation systems are usually equipped with low-precision inertial sensors and low-computing power processor. So a more efficient method should be proposed to offset the shortage of hardware.

For the traditional construction method, the vector in *ib* frame is calculated with $f^{b}$ and $\omega_{ib}^{b}$ by integrating from the initial moment to the current moment. According to (9), the output of accelerometers has been integrated once and the output of gyroscopes has been integrated twice. The double-vectors cannot be accurately calculated after the alignment has been implemented for a long time. For low-cost inertial sensors, the bias error cannot be ignored. It will accumulate a huge error with time and seriously affect the accuracy during the double-vectors construction [39]. The double-vectors are vitally important for the accuracy of the alignment because the optimal attitude is aiming to minimize the loss function constructed by the double-vectors. If the double-vector error is very large, the loss function will mislead the estimator to a wrong objective attitude, and it will even result in the failure of alignment. So the traditional method cannot meet the accuracy requirement of double-vectors for alignment.

For the part of the optimal estimation, the traditional method is very complicated and not applicable to the low-cost systems. Using Davenport’s method, the optimal quaternion can be obtained by finding the max eigenvalue of *K*, which is a matrix calculated with double-vectors. But it requires a significant amount of computing resources to calculate the eigenvalue and eigenvector. QUEST is derived from Davenport’s method to simplify the solution, which is the representative of traditional optimization-based alignment methods. The eigenvalues of *K* are the solution of the quartic equation. However, the computational burden of constructing and solving the quartic equation is also too heavy for low-cost processors.

The problem of the traditional OBA method can be mainly summarized as follows—(1) the double-vectors cannot be accurately constructed due to the error of low-cost inertial sensors; (2) the optimal quaternion or attitude matrix cannot be quickly obtained on the low-cost processors. Our research in this article is devoted to solving these problems.

## 3. The Proposed IFA Method

In this section, a new IFA method for the low-cost SINS/GPS integrated navigation system is investigated. This method has two parts—(1) The traditional construction method of double-vectors is modified to accurately construct the double-vectors with the measurement from low-cost inertial sensors. (2) A fast optimal attitude estimation method based on gradient descent is specially designed for low-cost systems. They are specified as follows.

### 3.1. Modified Construction Method of Double-Vectors

The error of double-vectors is mainly caused by the long-term integration of the bias of initial sensors according to the previous analysis. The construction method of double-vectors is modified to reduce the integration time of $f^{b}$ and identify the bias of gyroscope. Both α and β can be divided into two parts as

(21) $$\left\{ \begin{array}{l} \alpha \left(t\right)=\alpha \left(0,t_{m}\right)+\alpha \left(t_{m},t_{m+1}\right)\\ \beta \left(t\right)=\beta \left(0,t_{m}\right)+\beta \left(t_{m},t_{m+1}\right) \end{array}\right.,$$

where

(22) $$\begin{array}{l} \alpha \left(0,t_{m}\right)=\int_{0}^{t_{m}}C_{b\left(t\right)}^{ib}f^{b}dt\\ \alpha \left(t_{m},t_{m+1}\right)=\int_{t_{m}}^{t_{m+1}}C_{b\left(t\right)}^{ib}f^{b}dt=C_{b\left(t_{m}\right)}^{ib}\int_{t_{m}}^{t_{m+1}}C_{b\left(t\right)}^{b\left(t_{m}\right)}f^{b}dt \end{array}$$

and

(23) $$\begin{array}{l} \beta \left(0,t_{m}\right)=C_{n\left(t_{m}\right)}^{in}v^{n}\left(t_{m}\right)-v^{n}\left(0\right)+\int_{0}^{t_{m}}C_{n\left(t\right)}^{in}\left(2\omega_{ie}^{n}+\omega_{en}^{n}\right)\times v^{n}dt-\int_{0}^{t_{m}}C_{n\left(t\right)}^{in}g^{n}dt\\ \beta \left(t_{m},t_{m+1}\right)=C_{n\left(t_{m+1}\right)}^{in}v^{n}\left(t_{m+1}\right)-C_{n\left(t_{m}\right)}^{in}v^{n}\left(t_{m}\right)+\int_{t_{m}}^{t_{m+1}}C_{n\left(t\right)}^{in}\left(2\omega_{ie}^{n}+\omega_{en}^{n}\right)\times v^{n}dt-\int_{t_{m}}^{t_{m+1}}C_{n\left(t\right)}^{in}g^{n}dt \end{array}.$$

$\alpha \left(0,t_{m}\right)$ and $\beta \left(0,t_{m}\right)$ are the double-vector constructed from time 0 to time $t_{m}$, $\alpha \left(t_{m},t_{m+1}\right)$ and $\beta \left(t_{m},t_{m+1}\right)$ are the double-vector constructed from time $t_{m}$ to time $t_{m+1}$. Substituting (21) into (8) yields

(24) $$\alpha \left(0,t_{m}\right)+\alpha \left(t_{m},t_{m+1}\right)=C_{in}^{ib}\beta \left(0,t_{m}\right)+C_{in}^{ib}\beta \left(t_{m},t_{m+1}\right).$$

It can obviously be seen that $\alpha \left(0,t_{m}\right)=C_{in}^{ib}\beta \left(0,t_{m}\right)$ according to (8), so the relationship between modified double-vectors is as same as (8) and given by

(25) $$\alpha \left(t_{m},t_{m+1}\right)=C_{in}^{ib}\beta \left(t_{m},t_{m+1}\right).$$

For brevity, $\alpha \left(t_{m},t_{m+1}\right)$ and $\beta \left(t_{m},t_{m+1}\right)$ is hereafter omitted as α and β. In any same time interval, α and β can be converted to each other through $C_{in}^{ib}$ and they can be used for calculating the attitude matrix through the optimal estimation method. Compared to (9), the integration interval will not increase with time because the lower limit of integration of (22) can be an arbitrary moment before the current moment, which must be the initial time in (9). So the integration time of $f^{b}$ will not increase over time and the error caused by accelerometer bias can be reduced significantly.

However, the gyroscope measurement $\omega_{ib}^{b}$ which is used for calculating the attitude matrix $C_{b}^{ib}$ still needs to be integrated from the initial time. The calculated *ib* frame denoted by *ib’* is not coincident with the true *ib* frame due to the gyroscope bias denoted by $\varepsilon^{b}$ and the error angle between them is defined as $\varphi ={\left[\begin{array}{ccc} \varphi_{\theta } & \varphi_{\gamma } & \varphi_{\psi } \end{array}\right]}^{T}$ [40]. So the bias should be identified and feedback to the construction procedure. At the beginning of the alignment, the bias will not accumulate to a large error. The estimation attitude will close to the objective attitude and the misalignment angle can be regarded as a small angle. The Kalman filter is adopted to identify the gyroscope bias. According to liner inertial navigation system error equation, the simplified attitude error model is given by

(26) $$\dot{\varphi }=-C_{b}^{ib}\varepsilon^{b}.$$

Equation (26) is the process model of the Kalman filter. The equation of state transition is given by

(27) $$x_{k}=F_{k}x_{k-1}+w_{k-1},$$

where

(28) $$\begin{array}{l} x_{k}={\left[\varphi_{k},\varepsilon_{k}^{b}\right]}^{T}\\ F_{k}=\left[\begin{array}{cc} I_{3\times 3} & -C_{b}^{in^{\prime }}\\ 0_{3\times 3} & I_{3\times 3} \end{array}\right] \end{array}.$$

$x_{k}$ is the state of Kalman filter, *F_k_* is the state transition matrix and *w_k_* is the process noise. Denoting $\alpha^{\prime }$ as the vector observation in *ib’* frame and $\alpha =C_{ib^{\prime }}^{ib}\alpha^{\prime }$. The Equation (8) can be rewritten as

(29) $$C_{ib}^{in}\beta =C_{ib^{\prime }}^{ib}\alpha^{\prime }=\left(I_{3}+\left(\varphi \times \right)\right)\alpha^{\prime }.$$

According to (29), the measurement model of Kalman filter is given by

(30) $$\alpha^{\prime }-C_{in}^{ib}\beta =-\left(\varphi \times \right)\alpha^{\prime }=\left(\alpha^{\prime }\times \right)\varphi .$$

The equation of measurement can be written as

(31) $$z_{k}=H_{k}x_{k}+n_{k},$$

where

(32) $$\begin{array}{l} z_{k}=\alpha^{\prime }-C_{in}^{ib}\beta \\ H_{k}=\left[\begin{array}{cc} \alpha^{\prime }\times & 0_{3\times 3} \end{array}\right] \end{array}.$$

$z_{k}$ is the vector of measurement, $H_{k}$ is the transformation matrix linking the state vector and the measurement vector. $n_{k}$ is the measurement noise.

### 3.2. Fast IFA Method Mased on Gradient Descent

The double-vectors can be accurately obtained by the modified construction method mentioned above. For calculating the attitude matrix between the double-vectors, a fast IFA method based on gradient descent is proposed in this article. Denoting the Euler angle between *ib* frame and *in* frame as $A={\left[\begin{array}{ccc} \theta & \gamma & \psi \end{array}\right]}^{T}$ and $C_{in}^{ib}$ can be derived by *A* as follow

(33) $$\begin{array}{cc} C_{in}^{ib} & =C\left(\gamma \right)C\left(\theta \right)C\left(\psi \right)\\ & =\left[\begin{array}{ccc} \cos\gamma & 0 & -\sin\gamma \\ 0 & 1 & 0\\ \sin\gamma & 0 & \cos\gamma \end{array}\right]\left[\begin{array}{ccc} 1 & 0 & 0\\ 0 & \cos\theta & \sin\theta \\ 0 & -\sin\theta & \cos\theta \end{array}\right]\left[\begin{array}{ccc} \cos\psi & -\sin\psi & 0\\ \sin\psi & \cos\psi & 0\\ 0 & 0 & 1 \end{array}\right] \end{array}.$$

So $\beta =\left[\begin{array}{ccc} \beta_{x} & \beta_{y} & \beta_{z} \end{array}\right]$ can be converted to $\alpha =\left[\begin{array}{ccc} \alpha_{x} & \alpha_{y} & \alpha_{z} \end{array}\right]$ in three steps according to (25) and (33) as

(34) $$\beta \overset{C\left(\psi \right)}{\to }\beta_{1}\overset{C\left(\theta \right)}{\to }\beta_{2}\overset{C\left(\gamma \right)}{\to }\alpha ,$$

where $\beta_{1}$ and $\beta_{2}$ is the transition attitude matrix from β to α. The estimation of $C_{in}^{ib}$ is denoted by ${\tilde{C}}_{in}^{ib}$ and the estimation of α is $\tilde{\alpha }={\tilde{C}}_{in}^{ib}\beta$. The objective function of gradient descent is given by

(35) $$\begin{array}{cc} J\left(A\right) & =\sum_{k=1}^{N}\frac{1}{2N}{\left[{\tilde{\alpha }}_{k}-\alpha_{k}\right]}^{T}\left[{\tilde{\alpha }}_{k}-\alpha_{k}\right]=\sum_{k=1}^{N}\frac{1}{2N}\Delta \alpha_{k}^{T}\Delta \alpha_{k}\\ & =\sum_{k=1}^{N}\frac{1}{2N}\left(\Delta \alpha_{kx}^{2}+\Delta \alpha_{ky}^{2}+\Delta \alpha_{kz}^{2}\right) \end{array},$$

where $\Delta \alpha_{k}={\tilde{\alpha }}_{k}-\alpha_{k}$ which is a function of the attitude matrix *A.* The attitude angle θ, γ and ψ which can minimize the objective function J(A) is the solution of the IFA problem. The gradient equation objective function is given by

(36) $$\nabla J\left(A\right)=\left[\frac{\partial J\left(A\right)}{\partial \gamma },\frac{\partial J\left(A\right)}{\partial \theta },\frac{\partial J\left(A\right)}{\partial \psi }\right]=\sum_{k=1}^{N}\frac{1}{N}D\left(A\right)\Delta \alpha_{k}^{T},$$

where D(A) is the Jacobian matrix. ${\tilde{\alpha }}_{k}$ is the only term which contains attitude angle in $\Delta \alpha_{k}$, So D(A) can be written as follow

(37) $$D\left(A\right)=\left[\begin{array}{c} {\left(\frac{\partial \Delta \alpha_{k}}{\partial \gamma }\right)}^{T}\\ {\left(\frac{\partial \Delta \alpha_{k}}{\partial \theta }\right)}^{T}\\ {\left(\frac{\partial \Delta \alpha_{k}}{\partial \psi }\right)}^{T} \end{array}\right]=\left[\begin{array}{c} {\left(\frac{\partial {\tilde{\alpha }}_{k}}{\partial \gamma }\right)}^{T}\\ {\left(\frac{\partial {\tilde{\alpha }}_{k}}{\partial \theta }\right)}^{T}\\ {\left(\frac{\partial {\tilde{\alpha }}_{k}}{\partial \psi }\right)}^{T} \end{array}\right]=\left[\begin{array}{ccc} \frac{\partial {\tilde{\alpha }}_{kx}}{\partial \gamma } & \frac{\partial {\tilde{\alpha }}_{ky}}{\partial \gamma } & \frac{\partial {\tilde{\alpha }}_{kz}}{\partial \gamma }\\ \frac{\partial {\tilde{\alpha }}_{kx}}{\partial \theta } & \frac{\partial {\tilde{\alpha }}_{ky}}{\partial \theta } & \frac{\partial {\tilde{\alpha }}_{kz}}{\partial \theta }\\ \frac{\partial {\tilde{\alpha }}_{kx}}{\partial \psi } & \frac{\partial {\tilde{\alpha }}_{ky}}{\partial \psi } & \frac{\partial {\tilde{\alpha }}_{kz}}{\partial \psi } \end{array}\right],$$

where $\left[\begin{array}{ccc} \Delta \alpha_{kx} & \Delta \alpha_{ky} & \Delta \alpha_{kz} \end{array}\right]=\Delta \alpha_{k}$. $\frac{\partial {\tilde{\alpha }}_{k}}{\partial \gamma }$ is the first row of the Jacobian matrix and it represents the gradient of $\Delta \alpha_{k}$ in the direction of γ. According to (34), $\frac{\partial {\tilde{\alpha }}_{k}}{\partial \gamma }$ can be calculated as

(38) $$\frac{\partial {\tilde{\alpha }}_{k}}{\partial \gamma }=\frac{\partial \left(C\left(\gamma \right)\beta_{2}{}_{k}\right)}{\partial \gamma }=\left[\begin{array}{ccc} -\sin\gamma & 0 & -\cos\gamma \\ 0 & 0 & 0\\ \cos\gamma & 0 & -\sin\gamma \end{array}\right]\left[\begin{array}{c} \beta_{2kx}\\ \beta_{2ky}\\ \beta_{2kz} \end{array}\right]=\left[\begin{array}{c} -\sin\gamma \beta_{2kx}-\cos\gamma \beta_{2kz}\\ 0\\ \cos\gamma \beta_{2kx}-\sin\gamma \beta_{2kz} \end{array}\right],$$

where

(39) $$\begin{array}{cc} \beta_{2k} & =\left[\begin{array}{c} \beta_{2kx}\\ \beta_{2ky}\\ \beta_{2kz} \end{array}\right]=\left[\begin{array}{ccc} 1 & 0 & 0\\ 0 & \cos\theta & \sin\theta \\ 0 & -\sin\theta & \cos\theta \end{array}\right]\left[\begin{array}{ccc} \cos\psi & -\sin\psi & 0\\ \sin\psi & \cos\psi & 0\\ 0 & 0 & 1 \end{array}\right]\left[\begin{array}{c} \beta_{kx}\\ \beta_{ky}\\ \beta_{kz} \end{array}\right]\\ & =\left[\begin{array}{c} \cos\psi \beta_{kx}-\sin\psi \beta_{ky}\\ \cos\theta \sin\psi \beta_{kx}+\cos\psi \cos\theta \beta_{ky}+\sin\theta \beta_{kz}\\ \sin\theta \sin\psi \beta_{kx}+\cos\psi \cos\theta \beta_{ky}+\cos\theta \beta_{kz} \end{array}\right] \end{array}.$$

$\frac{\partial {\tilde{\alpha }}_{k}}{\partial \theta }$ can be calculated as

(40) $$\begin{array}{l} \frac{\partial {\tilde{\alpha }}_{k}}{\partial \theta }=\frac{\partial \left(C\left(\gamma \right)\beta_{k2}\right)}{\partial \beta_{k2}}\frac{\partial \beta_{k2}}{\partial \theta }=C\left(\gamma \right)\frac{\partial \left(C\left(\theta \right)\beta_{k1}\right)}{\partial \theta }=\left[\begin{array}{ccc} \cos\gamma & 0 & -\sin\gamma \\ 0 & 1 & 0\\ \sin\gamma & 0 & \cos\gamma \end{array}\right]\left[\begin{array}{ccc} 0 & 0 & 0\\ 0 & -\sin\theta & \cos\theta \\ 0 & -\cos\theta & -\sin\theta \end{array}\right]\left[\begin{array}{c} \beta_{1kx}\\ \beta_{1ky}\\ \beta_{1kz} \end{array}\right]\\ =\left[\begin{array}{c} \cos\theta \sin\gamma \beta_{1ky}+\sin\theta \sin\gamma \beta_{1kz}\\ -\sin\theta \beta_{1ky}+\cos\theta \beta_{1kz}\\ \cos\theta \cos\gamma \beta_{1ky}-\sin\theta \cos\gamma \beta_{1kz} \end{array}\right] \end{array}$$

where

(41) $$\beta_{1k}=\left[\begin{array}{c} \beta_{1kx}\\ \beta_{1ky}\\ \beta_{1kz} \end{array}\right]=\left[\begin{array}{ccc} \cos\psi & -\sin\psi & 0\\ \sin\psi & \cos\psi & 0\\ 0 & 0 & 1 \end{array}\right]\left[\begin{array}{c} \beta_{kx}\\ \beta_{ky}\\ \beta_{kz} \end{array}\right]=\left[\begin{array}{c} \cos\psi \beta_{kx}-\sin\psi \beta_{ky}\\ \sin\psi \beta_{kx}+\cos\psi \beta_{ky}\\ \beta_{kz} \end{array}\right].$$

$\frac{\partial {\tilde{\alpha }}_{k}}{\partial \psi }$ can be calculated as

(42) $$\begin{array}{l} \frac{\partial {\tilde{\alpha }}_{k}}{\partial \psi }=\frac{\partial \left(C\left(\gamma \right)C\left(\theta \right)\beta_{1k}\right)}{\partial \beta_{1k}}\frac{\partial \beta_{1k}}{\partial \psi }=C\left(\gamma \right)C\left(\theta \right)\frac{\partial \left(C\left(\psi \right)\beta \right)}{\partial \psi }\\ =\left[\begin{array}{ccc} \cos\gamma & 0 & -\sin\gamma \\ 0 & 1 & 0\\ \sin\gamma & 0 & \cos\gamma \end{array}\right]\left[\begin{array}{ccc} 1 & 0 & 0\\ 0 & \cos\theta & \sin\theta \\ 0 & -\sin\theta & \cos\theta \end{array}\right]\left[\begin{array}{ccc} -\sin\psi & -\cos\psi & 0\\ \cos\psi & -\sin\psi & 0\\ 0 & 0 & 0 \end{array}\right]\left[\begin{array}{c} \beta_{kx}\\ \beta_{ky}\\ \beta_{kz} \end{array}\right]\\ =\left[\begin{array}{c} -\left(\cos\gamma \sin\psi -\sin\gamma \sin\theta \cos\psi \right)\beta_{kx}-\left(\cos\gamma \cos\psi +\sin\gamma \sin\theta \sin\psi \right)\beta_{ky}\\ \cos\theta \cos\psi \beta_{kx}-\cos\theta \sin\psi \beta_{ky}\\ -\left(\sin\gamma \sin\psi +\cos\gamma \sin\theta \cos\psi \right)\beta_{kx}-\left(\sin\gamma \cos\psi -\cos\gamma \sin\theta \sin\psi \right)\beta_{ky} \end{array}\right] \end{array}.$$

The gradient of the objective function ∇J(A) can be obtained according to (36)–(42). The update equation of gradient descent is

(43) $$A_{i+1}=A_{i}-h\cdot \nabla J\left(A_{i}\right),$$

where h is the learning rate, which decides the size of the steps to approach the objective attitude. The subscript *i* denotes the *i*th estimation of attitude angle. The attitude angle will converge to the objective value along the opposite direction of the gradient through iteration. The parameter *N* in Equation (36) determines how many pairs of double-vectors are used for calculating the ∇J(A). The gradient descent method has many forms in terms of different batch sizes [41]. For the batch gradient descent (BGD), *N* is the number of the whole data and each update of the gradient will use all double-vectors. The attitude angle will be converged along the fastest direction of gradient descent to minimize the objective function, but this method is computationally intensive. For the stochastic gradient descent (SGD), only one random double-vectors is used for updating the gradient, so the computing burden is reduced. Each descent will not follow the fastest direction of whole data, so the fluctuation of the estimation process may be very severe. And it performs poor robustness in our practical use. Different from the above two methods, the mini-batch gradient descent (GDMBGD) use a part of the whole data for gradient update. It will take less time to update the gradient than BGD and have better robustness than SGD. The performance of MBGD will be different with different sizes of mini-batch data.

## 4. Simulation and Experiment Results

In this section, extensive simulations and experiments are implemented to verify and evaluate the performance of the proposed IFA method. Firstly, the simulation is carried out for comparing the proposed method with the traditional method. Secondly, a series of simulations are implemented to discuss the performance of the gradient descent for different sizes of mini-batch. Finally, a vehicle experiment verifies the proposed method is superior to the traditional method in practical use.

### 4.1. Simulation For the Proposed IFA Method

In this section, a simulation is carried out to compare the performance of the proposed IFA method and the traditional method represented by QUEST. The raw data of inertial and GPS data are generated by the simulator. The bias of three-axis accelerometers is set to 1mg and the bias of three-axis gyroscopes is set to 10°/h. The true pitch, roll and yaw angle are 20°, 40° and 60°, respectively. The learning rate of gradient descent is 0.2. In this simulation, the proposed BGD and modified construction method (MDCM) are compared with QUEST and the traditional double-vectors construction method.

**Figure 1:**
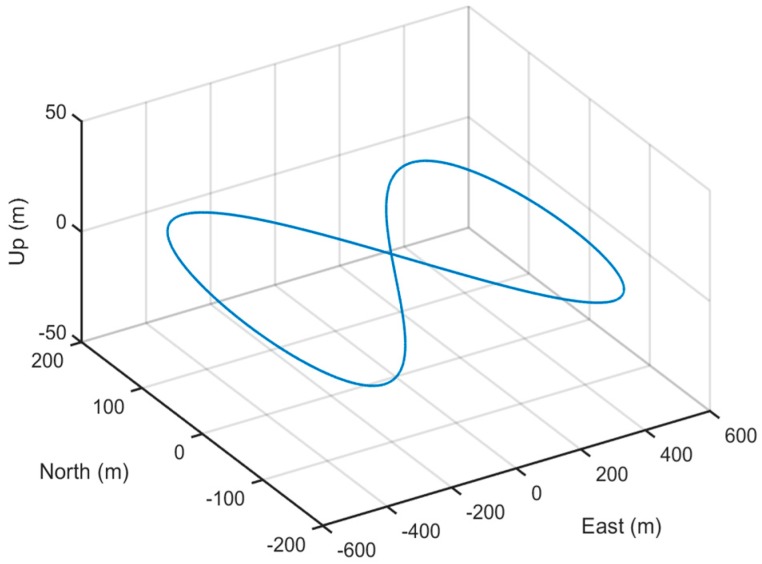
Simulation trajectory.

**Figure 2:**
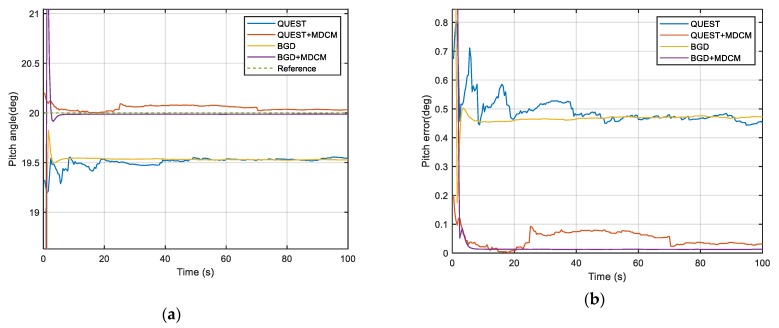
(**a**) Simulation results of pitch angle; (**b**) Simulation results of pitch error (absolute value).

**Figure 3:**
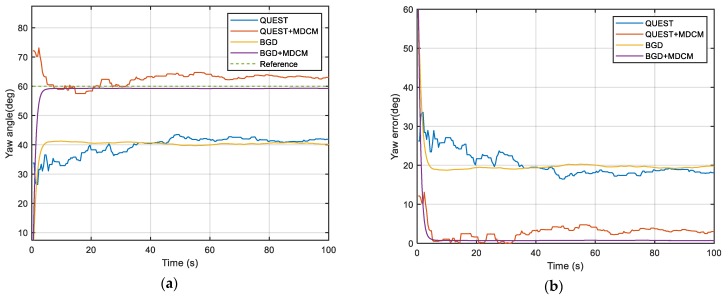
(**a**) Simulation results of roll angle; (**b**) Simulation results of roll error (absolute value).

**Figure 4:**
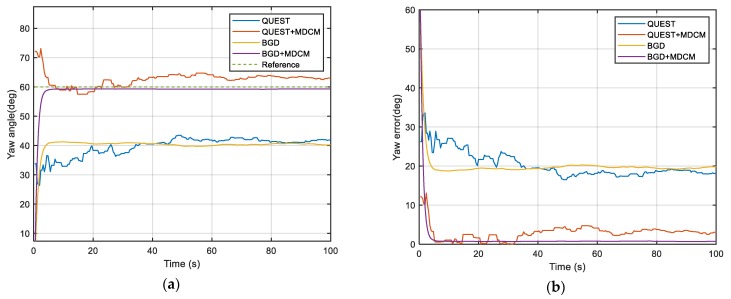
(**a**) Simulation results of yaw angle; (**b**) Simulation results of yaw error (absolute value).

The true simulation trajectory is shown in Figure 1 and the simulation results are shown in Figure 2, Figure 3 and Figure 4 and Table 1. The root-mean-square error (RMSE) is adopted to compare the accuracy of the alignment after convergence. It is defined as

(44) $$RMSE=\sqrt{\frac{1}{N}\sum_{i=1}^{N}{\left(A_{i}-{\hat{A}}_{i}\right)}^{2}}.$$

According to the results, it can be clearly seen that the attitude angle error of QUEST and BGD with the traditional method is very large. For the traditional double-vectors construction method, the bias error of inertial sensors will be accumulated over time and resulting in huge calculation errors of double-vectors. It will mislead the optimal estimator to a wrong objective value because the minimum point of the objective function is not at the objective attitude. So, the traditional construction method is not able to estimate the attitude angle accurately when the bias cannot be ignored. The performance of both two methods is obviously improved by MDCM because the inertial bias is identified and fed back to the procedure of double-vector construction, so the error is suppressed significantly. This simulation shows that the proposed MDCM is very effective and necessary for the alignment. It also can be seen that BGD has better performance than QUEST. The BGD is very stable after convergence. It has better precision and robustness. Due to the simplicity of the algorithm, the BGD takes less alignment time than QUEST.

### 4.2. Simulation For Different Sizes of Mini-Batch

**Figure 5:**
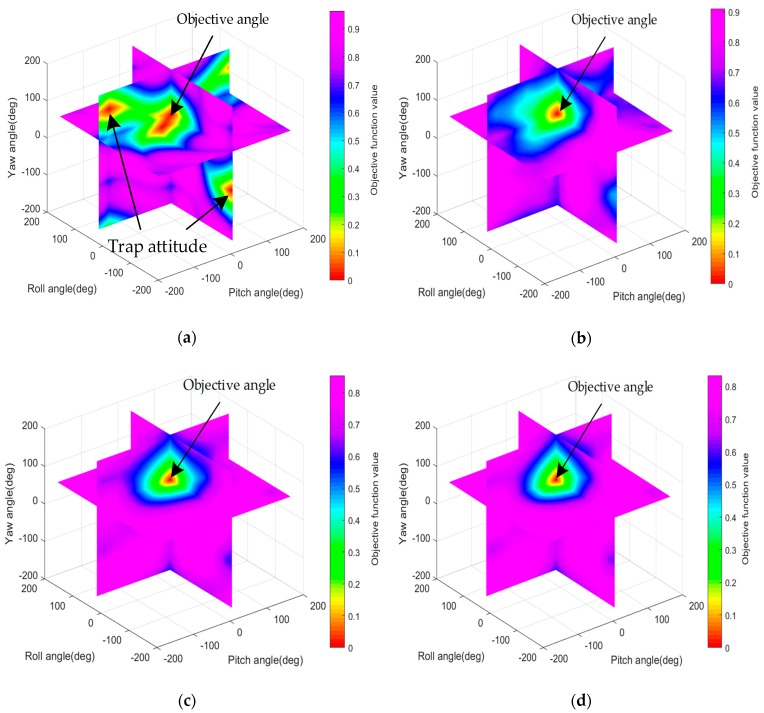
(**a**) Objective function calculated with one double-vector; (**b**) Objective function calculated with 10 double-vectors; (**c**) Objective function calculated with 60 double-vectors; (**d**) Objective function calculated with 1000 double-vectors.

In this section, the simulations are carried out to discuss the effect of different sizes of mini-batch on gradient descent performance. The objective function is the core of the gradient method. Because it determines the gradient and the objective attitude of the optimal estimation. The objective functions constructed with different sizes of mini-batch have different effects on the performance of gradient descent. The distinction can be visually seen through the four-dimensional graph of the objective function as shown in Figure 5.

Figure 5 from (a) to (d) are the objective functions which are respectively calculated with 1 double-vectors, one double-vectors, 10 double-vectors, 60 double-vectors and 1000 double-vectors. Three axes are respectively the pitch angle, roll angle and yaw angle of objective attitude. The color indicates the value of the objective function and the value of the objective function decreases as the hue warms. Three slices which are respectively at 20°, 40° and 60° of three-axis are selected to see the value of the function. The intersection of the three slices is the objective attitude angle. And it is the minimum point of the objective function in all four figures.

It can be clearly seen that as the mini-batch size increases, the gradient becomes steeper. It means the estimation process will be faster and more stable and the performance will be better. As shown in Figure 5a, the minimum point is not unique, the descent may not follow the direction of the objective point even all into the trap. So SGD needs more iterations to approach the lowest point of the objective function and the convergence process will fluctuate violently. In Figure 5b, the objective function is calculated with 10 pairs of double-vectors and there is only one minimum point which is the objective attitude. The direction of descent is clearer compared with Figure 5a. It can be seen from Figure 5c, whose function is calculated with 60 pairs of double-vectors and Figure 5d, whose function is calculated with 1000 pairs, that there is little difference between them. So The performance of MBGD which takes less computation is similar to that of BGD. Actually, the SGD and BGD can also regard as the MBGD with one and all double-vectors, in other word, mini-batch=1 and mini-batch=1000, respectively. So the key to the problem is to find a proper mini-batch to make the best performance for practical use.

SGD, MBGD and BGD are adopted for implementing the IFA simulation to compare their performance. The 10mg accelerometer bias noise and 10°/h gyroscope bias noise are added to the raw inertial data for simulating the measurement obtained by low-cost inertial sensors. The learning rate is set to 0.2. The initial estimation of the attitude angle can be arbitrary and is set to $A_{0}={\left[\begin{array}{ccc} 0 & 0 & 0 \end{array}\right]}^{T}$ in this simulation. The code is running on a computer of which CPU is Intel Core i5-10210@1.6GHz and the operating system is Windows 10.

**Figure 6:**
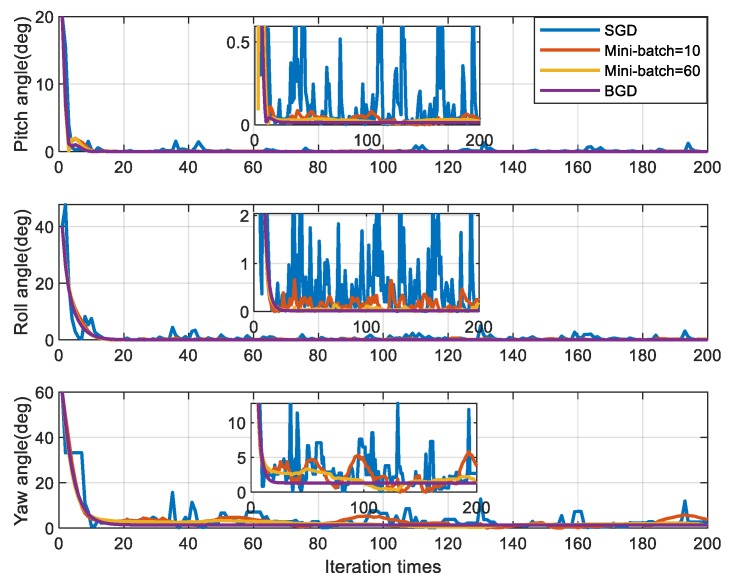
Simulation results of attitude angle.

**Figure 7:**
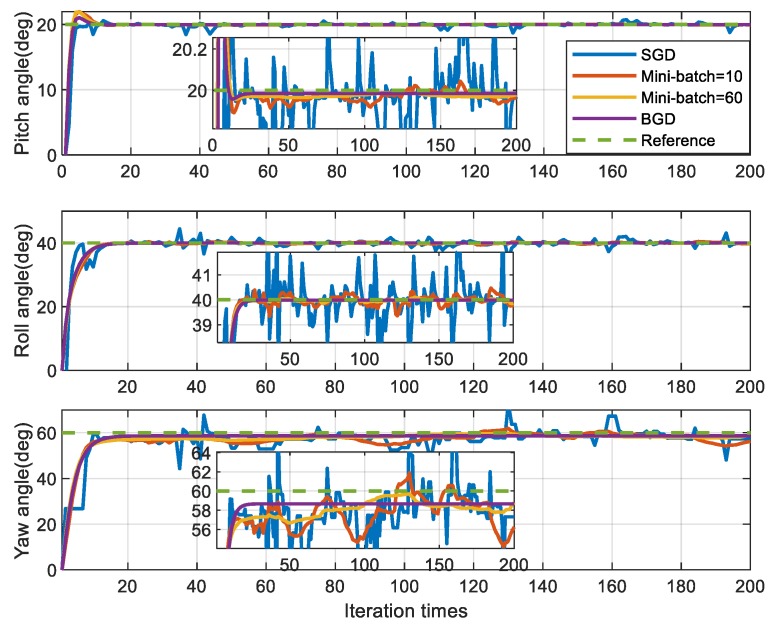
Simulation results of attitude angle error (absolute value).

Figure 6 and Figure 7 respectively show the simulation results of the IFA for four methods, which differ in the size of mini-batch. All four gradient descent methods are convergent near the objective attitude angle, but they have different performances. According to the Figure 6 and Figure 7 and Table 2, the attitude angle estimated by SGD fluctuates violently around the objective attitude and its error is the biggest among the four methods. For MBGD with mini-batch =10, the convergence is smoother and more accurate than SGD but there are still some fluctuations. When the mini-batch increased to 60, the performance of MBGD is improved significantly. BGD has the best accuracy and robustness and it is very stable near the objective attitude angle after convergence.

**Figure 8:**
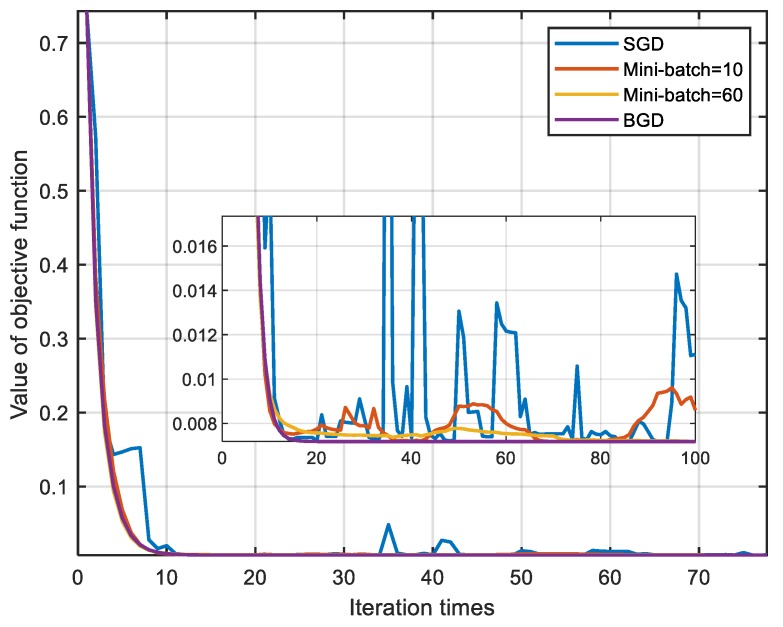
Simulation results of the objective function.

**Figure 9:**
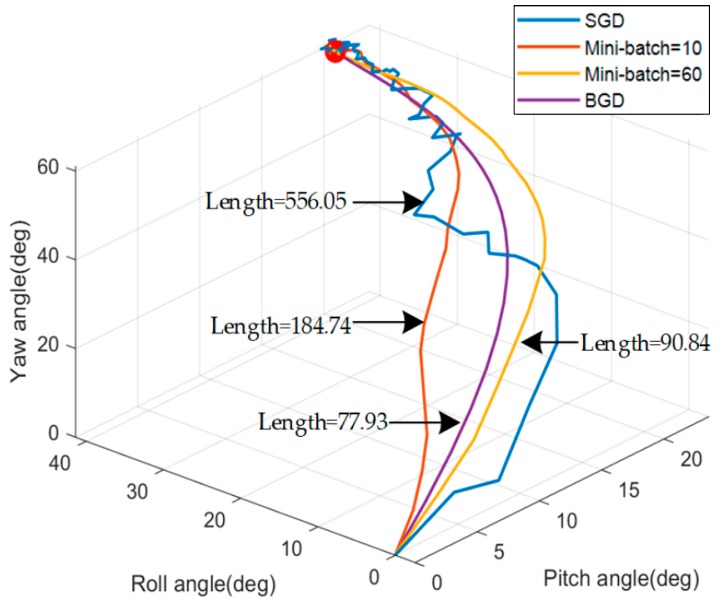
Simulation results of the gradient descent path.

Figure 8 is the descent curve of objective functions. The value of objective functions directly reflects the error of the attitude angle and all four methods can converge to a small value. It can be seen that as the number of double-vectors increases, the function converges more stably. Figure 9 is the descent path map of the four methods. The length of the path has no actual physical meaning and it is only used to compare the length of the descent process. The length of the path decreases with the increase of mini-batch. SGD has the longest path because the descent does not always follow the direction which is to the minimum point. BGD has the shortest path because each descent is in the right direction. However, different methods take different time for each descent. As shown in Table 2, the method whose mini-batch is bigger will cost more time to accomplish one attitude angle update.

According to the simulation result, there is not much difference in the number of iterations required for convergence. So the alignment will be faster with the size of the mini-batch reduce but the accuracy will also be reduced accordingly. The results of each update time and convergence time are measured. The mini-batch should be selected appropriately for different situations to balance computation speed and precision.

### 4.3. Experiment Results

**Figure 10:**
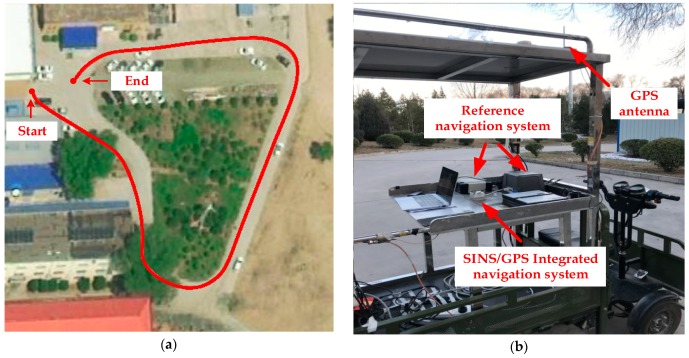
(**a**) Vehicle experiment trajectory; (**b**) Vehicle experiment platform.

In this section, the vehicle experiment is implemented as shown in Figure 10 to compare the performance of the proposed method and the traditional method. The SINS is composed of three gyroscopes of which bias is 1°/h (1σ) and three accelerometers of which bias is 1 mg (1σ), the position and velocity accuracy of GPS is 2 m (1σ) and 0.2 m/s (1σ) respectively. The vehicle used in the experiment is equipped with a high-precision navigation system produced by NovAtel, which can provide attitude (pitch and roll accuracy 0.008°, yaw accuracy 0.023°), velocity (accuracy 0.03 m/s) and position (accuracy 0.6 m) information as a reference for the alignment. The experiment was carried out at 112°26′44″ E, 38°0′51″ N (The North University of China, Taiyuan, Shanxi). The length of data is 100 s. The alignment starts at the 20 s and the objective attitude, which is the attitude angle between the *ib* frame and *in* frame at this moment, is respectively −3.79°, 3.12° and 160.24° according to the reference navigation system.

**Figure 11:**
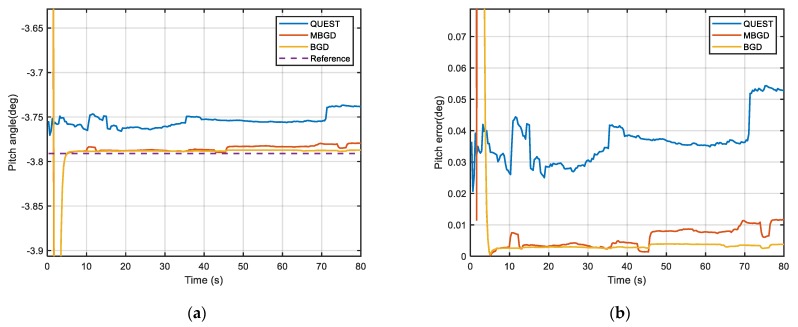
(**a**) Comparison of pitch angle; (**b**) Comparison of pitch error.

**Figure 12:**
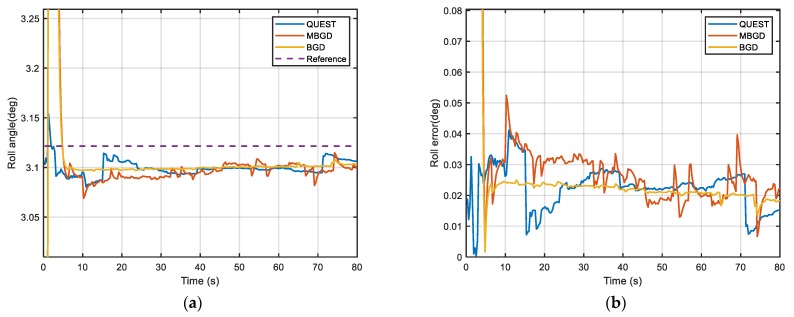
(**a**) Comparison of roll angle; (**b**) Comparison of roll error.

**Figure 13:**
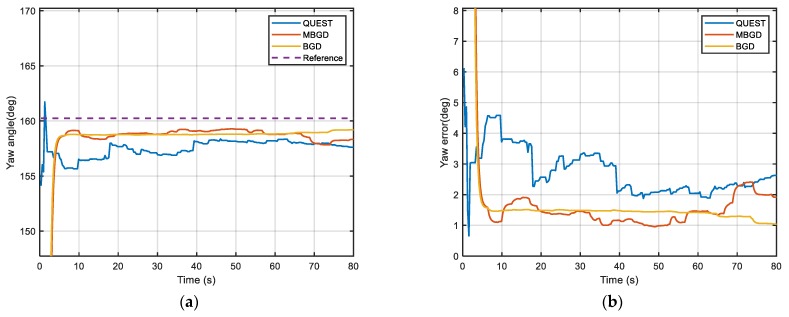
(**a**) Comparison of yaw angle; (**b**) Comparison of yaw error.

Figure 11, Figure 12 and Figure 13 show the alignment results of three different IFA methods, which are, respectively, QUEST, MBGD (mini-batch = 60) and BGD. They use the same set of data to estimate the optimal attitude angle. The performance of both two gradient descent methods is better than the traditional method QUEST. They have better accuracy and faster alignment time. Moreover, BGD is more robust in practical use. According to Figure 11, Figure 12 and Figure 13 and Table 3, MBGD and BGD perform similarly in this experiment. From the above simulation analysis, it can be known that each update of BGD will consume more computing resources than MBGD, because more double-vectors are used for calculating the gradient. However, the accuracy may be only improved slightly. But for the case that the signal of measurement is seriously disturbed, the BGD will be more robust. The measurement noise of GPS is also the main cause of alignment error. Using higher precision GPS can further improve the alignment accuracy.

## 5. Conclusions

From the simulations and experiments above, the proposed method is superior to the traditional OBA methods. Using the modified double-vector construction method, the bias error of the inertial sensor can be identified and suppressed. For the gyroscope with bias 10°/h and the accelerometer with bias 10mg, the maximum level attitude angle error can be reduced from 1.17° to 0.05° and the yaw angle error can be reduced from 20.34° to 0.705°. Using the proposed IFA method, the alignment time is reduced from 39.2 s to 7.6 s. The accuracy and alignment speed are significantly improved. The simulation results of the gradient descent show that the size of the mini-batch determines the performance of the algorithm. The accuracy and robustness are better as the mini-batch size is larger and the code runs faster as the mini-batch size is smaller. So the proposed method is very adaptable. The vehicle experiments show that compared with the QUEST, the maximum level attitude angle error is reduced from 0.037° to 0.021°, the yaw angle error is reduced from 2.69° to 1.41°,and the alignment time is reduced from 38.79 s to 6.08 s.

The advantages of the new IFA method proposed in this article can be summarized as follows:

1. The accuracy of double-vectors, which is an important prerequisite for the accurate attitude estimation, is improved by MDCM. The affect from the bias of inertial sensor is greatly reduced, so the double-vectors can be accurately constructed by this method on the low-cost navigation systems.
2. The proposed optimal method based on gradient descent is more flexible. And it can meet various needs by adjusting the size of mini-batch in practical use. This method is very simple and easy to implement. This method does not involve any multi-dimensional matrix operations in the whole optimal estimation process. So it will consume fewer computing resources than the traditional methods and it is very friendly to low-performance processors.
3. Our method has high accuracy and robustness. When the measurement contains bias of sensors and white noise, The proposed method has better performance than the traditional method represented by QUEST, which is proved by the results of simulations and experiments in this article.

## Figures and Tables

**Table 1:** Simulation results of four IFA methods.

Method	Errors(deg)			Alignment Time(s)
	Pitch	Roll	Yaw	
QUEST	0.493	0.824	20.34	39.20
QUEST+MDCM	0.050	0.055	2.95	37.85
BGD	0.462	1.17	20.01	8.08
BGD+MDCM	0.013	0.040	0.705	7.60

**Table 2:** Performance comparison of four methods.

Method	Errors(Degree)			Each Update Time ^1^ (ms)	Convergence Time ^1^ (ms)
	Pitch	Roll	Yaw		
SGD	0.138	0.553	2.848	0.08	3.9
MBGD(mini-batch = 10)	0.032	0.155	2.352	0.13	5.8
MBGD(mini-batch = 60)	0.025	0.038	1.756	0.27	12.7
BGD	0.014	0.015	1.334	2.05	88.3

^1^ “Time” means the code running time.

**Table 3:** Performance comparison of three methods in the experiment.

Method	Errors(Degree)			Alignment Time(s)
	Pitch	Roll	Yaw	
QUEST	0.037	0.022	2.69	38.79
MBGD(mini-batch = 60)	0.056	0.025	1.47	7.36
BGD	0.003	0.021	1.41	6.08

## References

[1] Zhang Y., Yu F., Gao W., Wang Y. (2018). An improved strapdown inertial navigation system initial alignment algorithm for unmanned vehicles. Sensors.

[2] Chang L., Qin F., Jiang S. (2019). Strapdown Inertial Navigation System Initial Alignment based on Modified Process Model. IEEE Sens. J..

[3] Tian X., Chen J., Han Y., Shang J., Nan L. (2017). Pedestrian navigation system using MEMS sensors for heading drift and altitude error correction. Sens. Rev..

[4] Hu H., Zhang J. Application of Hybrid Filtering Algorithm Based on Neural Network in INS/GPS Integrated Navigation System. Proceedings of the 2018 IEEE 4th International Conference on Control Science and Systems Engineering (ICCSSE).

[5] Liu Y., Fan X., Lv C., Wu J., Li L., Ding D. (2018). An innovative information fusion method with adaptive Kalman filter for integrated INS/GPS navigation of autonomous vehicles. Mech. Syst. Sig. Process..

[6] Zhang H., Li T., Yin L., Liu D., Zhou Y., Zhang J., Pan F. (2019). A Novel KGP Algorithm for Improving INS/GPS Integrated Navigation Positioning Accuracy. Sensors.

[7] Zhang Y. (2019). A Fusion Methodology to Bridge GPS Outages for INS/GPS Integrated Navigation System. IEEE Access.

[8] Kim Y., An J., Lee J. (2018). Robust navigational system for a transporter using GPS/INS fusion. IEEE Trans. Ind. Electron..

[9] Zhang G., Lu C., Li Y. Research on Initial Alignment Method of SINS with Improved CKF. Proceedings of the 2019 IEEE 3rd Information Technology, Networking, Electronic and Automation Control Conference (ITNEC).

[10] Li J., Xu J., Chang L., Feng Z. (2014). An Improved Optimal Method For Initial Alignment. J. Navig..

[11] Chang L., Li J., Chen S. (2014). Initial Alignment by Attitude Estimation for Strapdown Inertial Navigation Systems. IEEE Trans. Instrum. Meas..

[12] Li J., Li Y., Liuxs B. (2018). Fast fine initial self-alignment of INS in erecting process on stationary base. J. Navig..

[13] Lu J., Liang S., Yang L. (2018). Analytic coarse alignment and calibration for inertial navigation system on swaying base assisted by star sensor. IET Sci. Meas. Technol..

[14] Zhang W., Peng G., Yuan B., Wang P., Huo Z., Yang Z. Improved Maximum Likelihood Filter Based on UD Decomposition Algorithm and its Application in Transfer Alignment. Proceedings of the 2019 Chinese Control Conference (CCC).

[15] Wei X., Huang G.R., Lu H., Peng Z.Y., Hao S.Y., Xu M.Q. Marginal Reduced High-degree CKF and its Application in Nonlinear Rapid Transfer Alignment. Proceedings of the 2018 International Conference on Computer Information Science and Application Technology.

[16] Ding Z.J., Zhou H., Zhang S.F., Yang H.B., Cai H. (2017). Initial Self-Alignment Method for Inertial Platform on a Stationary Base. J. Astronaut..

[17] Xu Y., Zhou T. (2019). Research on In-Flight Alignment for Micro Inertial Navigation System Based on Changing Acceleration using Exponential Function. Micromachines.

[18] Wang D., Lv H., Jie W. (2017). In-flight Initial Alignment for Small UAV MEMS-based Navigation via Adaptive Unscented Kalman Filtering approach. Aerosol Sci. Technol..

[19] Wang D., Dong Y., Li Q., Wu J., Wen Y. (2018). Estimation of small uav position and attitude with reliable in-flight initial alignment for MEMS inertial sensors. Metrol. Meas. Syst..

[20] Shuster M.D. (1993). A survey of attitude representations. Navigation.

[21] Pei F., Wei X., Liang Q. A Fast Alignment Algorithm Based on Adaptive Quaternion Kalman Filter. Proceedings of the 2017 9th International Conference on Intelligent Human-Machine Systems and Cybernetics (IHMSC).

[22] Liu J., Zhao T. (2019). In-flight alignment method of navigation system based on microelectromechanical systems sensor measurement. Int. J. Distrib. Sens. Netw..

[23] Wu Y., Pan X. (2013). Velocity/Position Integration Formula Part II: Application to Strapdown Inertial Navigation Computation. IEEE Trans. Aerosp. Electron. Syst..

[24] Shuster M.D., Oh S.D. (1981). Three-axis attitude determination from vector observations. J. Guid. Control..

[25] Markley F.L. (1988). Attitude determination using vector observations and the singular value decomposition. J. Astronaut. Sci..

[26] Mortari D. (1998). Euler-q Algorithm for Attitude Determination from Vector Observations. J. Guid. Control Dyn..

[27] Wu M., Wu Y., Hu X., Hu D. (2011). Optimization-based alignment for inertial navigation systems: Theory and algorithm. Aerosp. Sci. Technol..

[28] Chang L., Li J., Li K. (2016). Optimization-based alignment for strapdown inertial navigation system: Comparison and extension. IEEE Trans. Aerosp. Electron. Syst..

[29] Xu X. (Xiang Xu), Xu X. (Xiaosu Xu), Zhang T., Wang Z. (2018). In-Motion Filter-QUEST Alignment for Strapdown Inertial Navigation Systems. IEEE Trans. Instrum. Meas..

[30] Wu Y., Pan X. (2013). Velocity/Position Integration Formula Part I: Application to In-Flight Coarse Alignment. IEEE Trans. Aerosp. Electron. Syst..

[31] Xu X., Xu D., Zhang T., Zhao H. (2019). In-Motion Coarse Alignment Method for SINS/GPS Using Position Loci. IEEE Sens. J..

[32] Wahba G. (1965). A least squares estimate of satellite attitude. SIAM Rev..

[33] Lerner G.M. (1978). Three-axis attitude determination. Spacecraft Attitude Determination and Control.

[34] Keat J. (1977). Analysis of Least-Squares Attitude Determination Routine DOAOP.

[35] Shuster M. Approximate algorithms for fast optimal attitude computation. Proceedings of the Guidance and Control Conference.

[36] Savage P.G. (1998). Strapdown inertial navigation integration algorithm design part 1: Attitude algorithms. J. Guid. Control Dyn..

[37] Xu Y. (2016). Research on several key techniques of MINS/GNSS integrated navigation system in the guided projectiles. Ph.D. Thesis.

[38] Bao J., Qiao X., Li D. (2017). Double-vector attitude determination algorithm improving coarse alignment accuracy of strapdown inertial navigation system for sea cucumber fishing device. Trans. Chin. Soc. Agric. Eng..

[39] Liu M., Gao Y., Li G., Guang X., Li S. (2016). An improved alignment method for the Strapdown Inertial Navigation System (SINS). Sensors.

[40] Chang L., Zha F., Qin F. (2017). Indirect Kalman Filtering Based Attitude Estimation for Low-Cost Attitude and Heading Reference Systems. IEEE/ASME Trans. Mechatron..

[41] Ruder S. (2016). An overview of gradient descent optimization algorithms. arXiv.

